# Whole genome sequences of 70 indigenous Ethiopian cattle

**DOI:** 10.1038/s41597-024-03342-9

**Published:** 2024-06-05

**Authors:** Wondossen Ayalew, Wu Xiaoyun, Getinet Mekuriaw Tarekegn, Rakan Naboulsi, Tesfaye Sisay Tessema, Renaud Van Damme, Erik Bongcam-Rudloff, Min Chu, Chunnian Liang, Zewdu Edea, Solomon Enquahone, Yan Ping

**Affiliations:** 1 Key Laboratory of Animal Genetics and Breeding on Tibetan Plateau, Ministry of Agriculture and Rural Affairs, Key Laboratory of Yak Breeding Engineering, Lanzhou Institute of Husbandry and Pharmaceutical Sciences, Chinese Academy of Agricultural Sciences, Lanzhou, 730050 P.R. China; 2 Institute of Biotechnology, Addis Ababa University, Addis Ababa P.O. Box 1176, Addis Ababa, Ethiopia; 3 Scotland’s Rural College (SRUC), Roslin Institute Building, University of Edinburgh, Edinburgh, EH25 9RG UK; 4 Childhood Cancer Research Unit, Department of Women’s and Children’s Health, Karolinska Institute, Tomtebodavägen 18A, 17177 Stockholm, Sweden; 5 Department of Animal Biosciences, Swedish University of Agricultural Sciences, 75007 Uppsala, Sweden; 6 Ethiopian Bio and Emerging Technology Institute, Addis Ababa, Ethiopia

**Keywords:** Animal breeding, Agriculture

## Abstract

Indigenous animal genetic resources play a crucial role in preserving global genetic diversity and supporting the livelihoods of millions of people. In Ethiopia, the majority of the cattle population consists of indigenous breeds. Understanding the genetic architecture of these cattle breeds is essential for effective management and conservation efforts. In this study, we sequenced DNA samples from 70 animals from seven indigenous cattle breeds, generating about two terabytes of pair-end reads with an average coverage of 14X. The sequencing data were pre-processed and mapped to the cattle reference genome (ARS-UCD1.2) with an alignment rate of 99.2%. Finally, the variant calling process produced approximately 35 million high-quality SNPs. These data provide a deeper understanding of the genetic landscape, facilitate the identification of causal mutations, and enable the exploration of evolutionary patterns to assist cattle improvement and sustainable utilization, particularly in the face of unpredictable climate changes.

## Background & Summary

Indigenous animal genetic resources, primarily found in developing countries, are known to contain a significant portion of the world’s genetic diversity. Millions of people rely directly on these resources for their livelihoods^1^. Ethiopia, in particular, is considered a gateway for cattle migrations in Africa^2^. Presently, the cattle population in Ethiopia exceeds 70 million heads^3^, with 98.5% of them being indigenous cattle. These indigenous cattle are often named based on their appearance, morphological structure, the ethnic group of the herder, and their geographical location^4,5^. Over time, these cattle have developed unique adaptive traits that enable them to withstand challenges such as limited feed availability, high environmental temperatures, and a high prevalence of internal and external parasites and diseases. These adaptive features have been shaped through natural and human selection processes^6,7^.

By far, cattle production in Ethiopia is an integral part of almost all farming systems in the crop-livestock mixed farming systems of highlanders and mid attitudes, and the main occupation in the lowland pastoralists, and still promising to rally around the country’s economic development. Despite multiple functions and significant phenotypic variations of indigenous cattle populations, little attention was paid to the livestock sector, which threatened the country’s cattle diversity and population size. These are mainly associated with complex and interrelated factors such as indiscriminate crossbreeding and interbreeding between adjacent indigenous breeds due to herders’ migrations and socio-cultural interactions^8,9^. Furthermore, recurrent drought, the prevalence of disease, ethnic conflicts, and the illegal cross-border market hasten the decline in cattle numbers. Thus, a comprehensive understanding of breed characteristics, including population size, genetic landscape, and geographical distribution, is crucial for effectively managing farm animal genetic resources^1,10^. It also serves as a guiding framework for breed development programs, enabling them to align with specific production needs in diverse environments.

Quantitative genetic analysis has historically been characterized as a black box due to the intricate nature of gene action, which involves multiple loci with unknown effects and their interactions in shaping quantitative traits^11^. This complexity has posed challenges in understanding the underlying mechanisms and unraveling the genetic architecture of these traits. As a result, researchers have faced difficulties replicating the results of selective breeding across different spatial and temporal scales, making it essential to explore further and elucidate these complex genetic processes. Advancements in genome sequencing, SNP genotyping technologies, and statistical analysis tools have shifted research focus from analyzing neutral variation to exploring functional variation^12^. Notably, the advent of whole-genome sequencing (WGS) in domestic animals has revolutionized our understanding of their genetic makeup. It has allowed for the identification of causal variants that have significant implications for animal production, health, welfare, and evolutionary studies within livestock species and breeds^13^. While WGS has become a standard tool in various biological sciences, including animal breeding, its application for genetic characterization and routine evaluation of livestock genetic resources in developing countries is still limited. This study presents the whole-genome sequencing data from 70 indigenous cattle originating from seven distinct Ethiopian cattle populations sampled from various agro-ecological and climatic settings (Table 1; Ayalew *et al*.^14^). Thus, our WGS data will serve as a valuable resource for conducting further in-depth studies and investigations in tropical cattle. This sequence dataset will facilitate a deeper understanding of the genetic landscape, allowing for the identification and validation of causal mutations that contribute to essential traits and the exploration of evolutionary patterns.

**Table 1:** Ethiopian cattle breeds and their respective sampling locations.

Breeds	No. of samples	Geographic region	Altitude	Latitude	Longitude	Agro-Ecology
Abigar	10	Gambela	523	8.123469	34.30687	Hot, humid, and low-altitude
Barka	10	Amhara	895	14.18467	36.89087	Hot, humid, and low-altitude
Boran	10	Oromiya	1368	4.978936	38.27516	Hot, humid, and low-altitude
Felata	10	Amhara	552	12.40733	35.87573	Hot, humid, and low-altitude
Fogera	10	Amhara	1735	11.86045	37.81373	Humid and mid altitude
Gojjam-Highland	10	Amhara	3410	10.72113	37.85988	Cold, humid, and high-altitude
Horro	10	Oromiya	1722	9.672949	37.07545	Humid and mid-altitude

Moreover, the detailed analytical procedures offer significant advantages for researchers, such as ease of management of similar WGS and implementation of global cattle meta-assemblies at a broader scale. The meta-assembly, which combines multiple genetic or genomic data assemblies into a single, comprehensive assembly, will enable the accurate validation of regions under selection reported by various researchers, ensuring the identification of actual signals while minimizing false positives and supporting future breed improvement and conservation efforts.

## Methods

### Cattle sampling and collection

We specifically selected seven indigenous cattle populations (Abigar, Barka, Boran, Fellata, Fogera, Gojjam-Highland, and Horro) for our study, with ten unrelated samples collected from each population. These cattle populations inhabit distinct agro-climatic regions, representing Ethiopia’s diverse environments (Table 1). We selected these particular populations based on their relevance to agricultural practices, providing insights into desirable production traits, environmental adaptation, and regional livestock farming systems. Blood samples were drawn from the jugular vein of the cattle under sterile conditions, using 10 ml EDTA tubes. The samples were carefully transported to the laboratory in an ice box and stored at −20 °C until DNA extraction.

### Extraction and quality control of genomic DNA

The blood samples were thawed for 30 minutes at room temperature and underwent DNA extraction using the Tiangen genomic DNA extraction kit based on the manufacturer’s protocols (TIANGEN Biotech, Beijing, China). We conducted 0.8% agarose gel electrophoresis to assess DNA integrity and visualized the resulting DNA bands using a gel imaging apparatus. Each sample’s DNA concentration and quality were determined using a Nanodrop Spectrophotometer (ND-2000, Thermo Scientific, Massachusetts, USA) at a wavelength of A260/A280. Samples with DNA concentrations above 50 μg/μl were then sent to Wuhan Frasergen Bioinformatics Co. Ltd in China for whole-genome sequencing (WGS).

### Sequence library preparation and sequencing

The VAHTS Universal DNA Library Prep Kit for MGI (Vazyme, Nanjing, China) was employed to generate sequencing libraries of each sample, targeting fragments of approximately 500 bp in length using one microgram of DNA as input material. Adapter sequences were ligated to each sample. Library size and quantification were assessed using Qubit 3.0 Fluorometers and Bioanalyzer 2100 systems (Agilent Technologies, CA, USA). Finally, the sequencing process was conducted by Frasergen Bioinformatics Co., Ltd. (Wuhan, China) on an MGI-SEQ 2000 platform, resulting in a 150 bp sequence length for each sample.

### Sequence data pre-processing and mapping

The demultiplexed 70 individual samples (forward and reverse reads) were received and checked for their quality metrics using FastQC v0.11.8^15^. The raw reads were subjected to initial quality control by Trimmomatic v0.39 using default settings^16^. After removing adapter sequences and low-quality reads, MultiQC v1.14 was run on the clean reads, and standard sequence quality metrics were confirmed for subsequent analysis. BWA-MEM 0.7.17-r1188^17^ was employed to align individual reads to the latest bovine reference genome ARS-UCD1.2^18^. The aligned reads were converted to binary alignment map (BAM) format, sorted by coordinates, and indexed using SAMtools version 1.6^19^. Finally, the duplicate sequences were marked using the MarkDuplicates function of Picard 2.27.4 (https://broadinstitute.github.io/picard/) to produce a non-duplicated bam file for variant calling.

### Variant calling and filtration

**Fig. 1:**
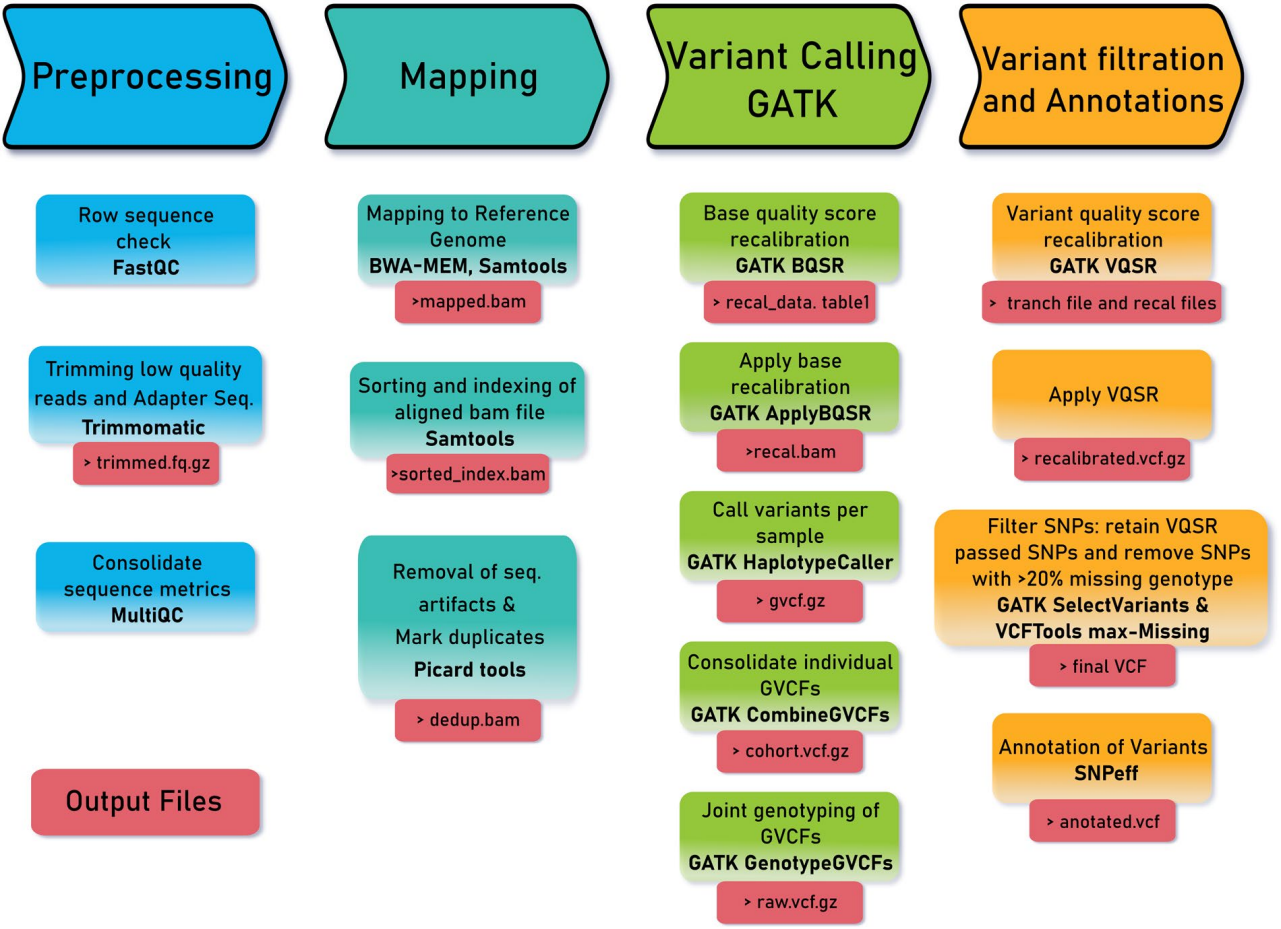
Overview of raw data quality control, sequence mapping, variant calling, and variant filtration pipeline. The pipeline follows GATK’s best practice protocol for germline short variant discovery.

High-quality variant calling and filtration are vital in genomic research. The Genome Analysis Toolkit best practices pipeline (https://gatk.broadinstitute.org/hc/en-us/articles/360035535932-Germline-short-variant discovery) was employed for SNPs discoveries (Fig. 1). First, the marked duplicate bam files were used as input to generate Base Quality Score Recalibration (BQSR) tables using GATK 4.3.0.0. The “Apply BQSR” argument of the same software was then employed to create recalibrated BAM files. The HaplotypCaller method, followed by joint genotyping of all samples and VQSR procedures for SNP recalibrations, was performed using validated SNPs provided by the 1000 bull genome project. In the Variant Quality Score Recalibration (VQSR) procedure, SNP recalibrations utilized different variant annotators, including Quality of Depth (Q.D.), Fisher Strand Test (F.S.), Mapping Quality Score (M.Q.), Mapping Quality Rank Sum Test (MQRankSum), Read Position Rank Sum Test Statistic (ReadPosRankSum), and StrandOddsRatio Test (SOR). Subsequently, the ApplyVQSR procedure was employed to select variants with a true sensitivity of 99.0%. Finally, the ‘SelectVariant’ procedure from the same software was used, and the final SNPs were used for annotations (refer to the Code availability section).

## Data Records

The 70 Ethiopian indigenous cattle pair-end raw sequencing data (in fastq.gz format) were available at NCBI under Sequence Read Archive (SRA) accession numbers SRP478348^20^ and SRP480803^21^ (Supplementary file 1). The VCF file can be available in the European Variation Archive (EVA) with the accession number for Project PRJEB75238 (https://identifiers.org/ena.embl:ERP159827)^22^.

## Technical Validation

### Quality control for raw reads and alignments

**Fig. 2:**
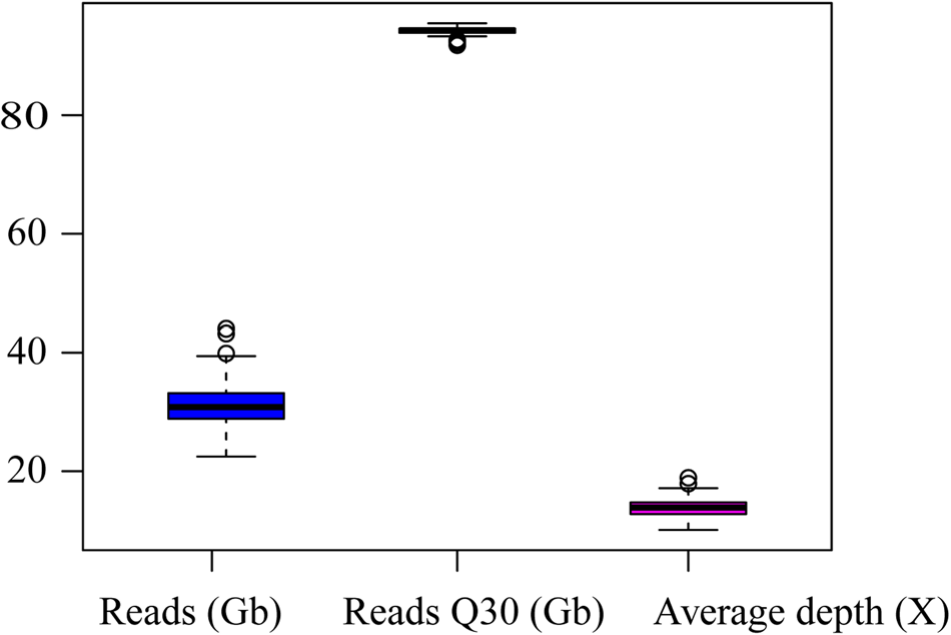
Boxplot presentation of 70 Ethiopian cattle sequencing yield, yield Q30 and estimated sequence coverage.

In next-generation sequencing (NGS) data analysis, quality control of raw sequence reads is a standard preliminary procedure before further analysis. This crucial pre-processing step enhances the overall data quality and reliability before conducting downstream analyses^23^. Some essential quality measures used to make choices for the downstream analysis are the base quality, nucleotide distribution, G.C. content, and duplication rate of the raw sequences^24^. Sequencing of each individual yielded between 13.61 gigabases to 25.45 gigabases, of which 91.8–95.5% of the reads fell above Phred scaled quality score of 30, which proves the bases were called with 99.9% accuracy (Fig. 2). To elucidate all types of variants (including SNVs, indels, and CNVs), a high-depth WGS (30X) is the ‘gold standard’^25^. Due to budget constraints, it is common practice to sequence fewer samples at high coverage (20 to 30X). However, this approach may result in a poor representation of a population’s genetic variation. The smaller dataset may not adequately capture the full range of genetic diversity present, leading to potential biases or incomplete insights^23^. Recently, Jiang *et al*. suggested 4X as the lowest boundary and 10X as an ideal depth for achieving greater than 99% genome coverage in pigs^26^. The average estimated coverage for each of the 70 Ethiopian cattle samples was above the threshold with an average depth of 14X (Fig. 2). The relatively moderate depth of coverage in our study enhances the resolution and reliability of downstream analyses, leading to more robust findings and insights into the genetic basis of various traits and population dynamics^26,27^.

**Fig. 3:**
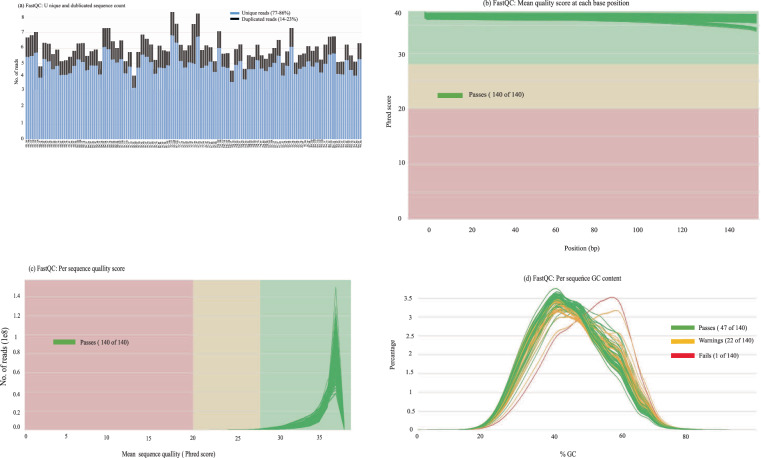
The quality control metrics from FastQC analysis of 70 cattle sequences. The metrics from all FASTQ files are consolidated using the MultiQC package.

The MultiQC software^28^ was employed to generate a pooled sequence quality metrics report (Fig. 3). The MultiQC reports for 70 paired-end Ethiopian cattle sequences confirm that the mean quality scores and per-sequence metrics fell within the high sequence standard range for downstream analysis (Fig. 3b,c). Although there is no universal threshold for duplication levels in WGS data, FastQC flagged a warning for sequences with more than 20% duplicates^15^. Unlike PCR-free methods, PCR-based sequencing introduces bias in sequencing data by causing uneven amplification of genomic regions and generating duplicate reads, which can impact the accuracy of the sequencing data^29^. Intriguingly, we found an average duplication rate of 17% (Fig. 3a), and this relatively low level of duplication observed in our data can mitigate challenges in variant calling and uneven distribution of coverage across the genome and enhance the efficiency and speed of analysis pipelines^30^.

A uniform G.C. content among reads indicates high-quality sequencing, suggesting minimal artifacts or contaminants^24^. However, in our dataset comprising 70 forward and 70 reverse sequencing files (140 files), all sequenced in the same lane and on the same instrument, Fig. 3d reveals some deviations from the expected distribution of G.C. content in a subset of 23 files (16.43%). These deviations may be attributed to challenges during library preparations^15^. Notably, despite deviations observed in the G.C. content distribution of some sequencing files, a warning message is acceptable for fewer than 30% of the reads, indicating that the overall data quality remains suitable for subsequent analysis^15^.

While the quality control for aligned reads is not routinely conducted, it is a valuable tool for gaining additional insights into sample quality. It can help identify problematic samples that might pass the initial raw data quality control checks^24^. In our data, 99.2% of the reads were successfully mapped to the *Bos taurus* (ARS-UCD1.2) reference genome (Supplementary file 2). It suggests that most reads were mapped correctly to their corresponding genomic locations.

### Quality control for SNP data

After consolidating individual sample VCF files, the joint genotyping analysis yielded 39 million SNPs. To ensure the reliability of these variants and filter out false-positive calls for downstream analyses, we employed a robust machine-learning model called VQSR (https://gatk.broadinstitute.org/hc/en-us/articles/360035531612-Variant-Quality-Score-Recalibration-VQSR). VQSR is a two-step process that involves training a machine learning model using a training dataset and then applying this model to recalibrate the variant quality scores in the primary dataset. VQSR offers several advantages, including improved accuracy, adaptability, comprehensive assessment, and reduced false positives compared to traditional filtering methods. By incorporating VQSR, we optimized the quality control process and enhanced the validity of our variant calls. Specifically, threshold values of 99% retained about 35 million true variants and excluded four million variants as poor/false positive calls. We also computed the transition/transversion (Ti/Tv) ratio and the heterozygosity-to-homozygosity (het/hom) ratio for SNPs passing the 99% threshold. The observed Ti/Tv and het/hom ratios were 2.35 and 1.17, respectively. These metrics are consistent with values reported for other African zebu cattle breeds^31^.

To investigate the genomic distribution and functional impact of genetic variants, we used the SNPeff variant annotation tool. A significant portion of variants (over 89%) were annotated within intronic and intragenic regions (Table 2). Notably, while the number of SNPs per chromosome correlated with chromosome length^32^, our study revealed varying SNP densities across chromosomes. For instance, Chromosome 23 showed the highest SNP density (16.65), whereas the X chromosome had the lowest (6.61). These variations are likely attributed to multiple factors, including differences in recombination and mutation rates, genetic drift, demographic influences, selective pressures, and population history^33^. Despite containing more repetitive regions, the X chromosome experiences heightened selection pressure against genetic variants, driven by hemizygosity in males and X-chromosome inactivation in females. As a result, the X chromosome exhibits a lower SNP density than autosomes. These unique genetic mechanisms and evolutionary dynamics significantly shape the distinct SNP profiles observed between the X chromosome and autosomes^34^ Table 3.

**Table 2:** Single Nucleotide Polymorphisms (SNPs) across various annotation categories.

Annotation categories	Count	% of total
Downstream	2,563,798	4.51%
Exon	513,998	0.90%
Intergenic	23,537,404	41.41%
Intron	27,406,871	48.22%
Splice_site_acceptor	613	0.00%
Splice_site_donor	966	0.00%
Splice_site_region	49,852	0.09%
Transcript	551	0.00%
Upstream	2,507,622	4.41%
UTR_3_prime	176,834	0.31%
UTR_5_prime	75,531	0.13%

**Table 3:** Summary of SNPs density in each chromosome.

BTA	CHR Length	SNP count	Density/kb
1	158534110	2225913	14.04
2	136231102	1835540	13.47
3	121005158	1571987	12.99
4	120000601	1692789	14.11
5	120089316	1578815	13.15
6	117806340	1653802	14.04
7	110682743	1467141	13.26
8	113319770	1509341	13.32
9	105454467	1442407	13.68
10	103308737	1391180	13.47
11	106982474	1437389	13.44
12	87216183	1312516	15.05
13	83472345	1092309	13.09
14	82403003	1126064	13.67
15	85007780	1265285	14.88
16	81013979	1116393	13.78
17	73167244	1042961	14.25
18	65820629	874411	13.28
19	63449741	847878	13.36
20	71974595	1041114	14.47
21	69862954	968519	13.86
22	60773035	836115	13.76
23	52498615	874180	16.65
24	62317253	916025	14.70
25	42350435	605379	14.29
26	51992305	747549	14.38
27	45612108	723378	15.86
28	45940150	726207	15.81
29	51098607	803719	15.73
X	139009144	919141	6.61
Unplaced	76654434	213849	2.79

### Supplementary information

Supplement File 1 and Supplement File 2

## Data Availability

Data analyses were primarily conducted using standard bioinformatics tools on the Linux operating system. We provide detailed information about the versions and code parameters of the software tools used at https://github.com/WondossenA/WGS_Ethiopian_cattle/blob/main/code_explanation.md.
